# T_1_ relaxation of bound and pore water in cortical bone

**DOI:** 10.1002/nbm.4878

**Published:** 2022-12-13

**Authors:** Thammathida Ketsiri, Sasidhar Uppuganti, Kevin D. Harkins, Daniel F. Gochberg, Jeffry S. Nyman, Mark D. Does

**Affiliations:** 1 Biomedical Engineering, Vanderbilt University, Nashville, Tennessee, USA; 2 Vanderbilt University Institute of Imaging Science, Vanderbilt University Medical Center, Nashville, Tennessee, USA; 3 Orthopaedic Surgery, Vanderbilt University Medical Center, Nashville, Tennessee, USA; 4 Veterans Affairs, Tennessee Valley Healthcare System, Nashville, Tennessee, USA; 5 Center for Bone Biology, Vanderbilt University Medical Center, Nashville, Tennessee, USA; 6 Radiology & Radiological Sciences, Vanderbilt University Medical Center, Nashville, Tennessee, USA; 7 Physics and Astronomy, Vanderbilt University, Nashville, Tennessee, USA; 8 Electrical Engineering, Vanderbilt University, Nashville, Tennessee, USA

**Keywords:** bone, NMR, pore water, relaxometry, T_1_

## Abstract

MRI measures of bound and/or pore water concentration in cortical bone offer potential diagnostics of bone fracture risk. The transverse relaxation characteristics of both bound and pore water are relatively well understood and have been used to design clinical MRI pulse sequences to image each water pool quantitatively. However, these methods are also sensitive to longitudinal relaxation characteristics, which have been less well studied. Here, spectroscopic relaxometry measurements of 31 human cortical bone specimens provided a more detailed picture of *T*_1_ of both bound and pore water. The results included mean, standard deviation, and range of *T*_1_ spectra from both bound and pore water, as well as novel presentations of the 2D *T*_1_ − *T*_2_ distribution of pore water. Importantly, for each sample the pore water *T*_1_ spectrum was found to span more than one order of magnitude and varied substantially across the 31 sample studies. Because many existing methods assume pore water *T*_1_ to be mono-exponential and constant across individuals, the results were used to compute the potential effect neglecting this intra- and intersample *T*_1_ variation on accurate MRI measurement of both bound and pore water concentrations. The greatest effect was found for adiabatic inversion recovery (AIR) based measurements of bound water concentration, which showed an average of 8.8% and as much as 37% error when using a common mono-exponential assumption of pore water *T*_1_. Despite these errors, the simulated AIR measurements were still moderately well correlated with the bound water concentrations derived from the spectroscopic data.

## 1 | INTRODUCTION

Osteoporosis is a worldwide health problem in which bone fractures lead to chronic pain, disability, and reduced life expectancy.^1^ The standard diagnostic tool for osteoporosis is dual energy X-ray absorptiometry, which provides areal measures of bone mineral density. However, mineral content alone is insufficient at predicting an individual’s bone fracture risk, which is also influenced by factors such as age and disease.^2–4^ Alternatively, MRI is sensitive to the soft tissue components of bone, such as the hydrated collagen matrix and pore spaces, and can provide a more complete evaluation of its changes at the microscopic scale. The MRI signals from cortical bone are primarily derived from (1) water in the vascular–lacunar–canalicular spaces (pore water, PW) and (2) collagen-bound water trapped within the bone matrix (bound water, BW).^5–7^ These signals can be distinguished by the transverse relaxation characteristics—BW *T*_2_ is quite short, ≈ 400 *μ*s, while PW *T*_2_ can be tens or hundreds of milliseconds. Numerous experimental studies have shown that absolute, relative, or total measures of BW and PW signal amplitudes predict a variety of bone mechanical properties such as yield stress, peak stress, and preyield toughness,^8–16^ but technical challenges remain for translation of this tool to clinical MRI.

Ultrashort echo time (UTE) MRI enables imaging the short-*T*_2_ signals in cortical bone^17^ on clinical systems, and in various forms offers the potential to improve diagnostics of bone health.^18,19^ One approach to measure BW or PW with UTE-MRI involves *T*_2_-selective adiabatic RF pulse preparations to suppress signal from one pool in order to image the other signal directly.^20–23^ These methods are known as adiabatic inversion recovery (AIR) and double adiabatic full passage (DAFP), for BW and PW measurements, respectively. Another approach is to acquire multiple images with varied $T_{2}^{*}$ weighting and fit these signals to a bi- or tricomponent signal model, which includes BW and PW terms.^10,24–26^ Both of these approaches rely on *T*_2_ or $T_{2}^{*}$ differences between BW and PW, but accurate results, particularly for the methods involving an inversion-recovery preparation, depend on some knowledge of the *T*_1_ characteristics of BW and PW.

In contrast to *T*_2_, information about the *T*_1_ characteristics of cortical bone is inconsistent across studies, for both total bone measurement and individual bone water-component measurements. In vivo, total water *T*_1_ measurements vary greatly, even those made from the same anatomical location. For example, from the tibia, various studies using different methods have reported *T*_1_ = 140–260 ms,^27^ 74–103 ms,^28^ 100–350 ms,^29^ and 209–229 ms.^30^ To some extent, this variation may reflect varying sensitivities of each measurement protocol to the BW and PW components.

For the purpose of quantitative imaging of BW and/or PW, component-specific *T*_1_ measurements are needed, and literature on these values is limited and also varied. In vivo at 3T, Chen et al. measured *T*_1,bw_ = 134±11ms (mean ± standard deviation) and *T*_1,pw_ = 524±46ms from the tibia of eight healthy young male volunteers.^31^ At 1.5T, over a wider age range, and including both male and female subjects, Akbari et al. reported *T*_1,pw_ = 100–250ms.^32^ Similar results, with somewhat longer *T*_1_ values, were reported from studies at 3.0T.^33^ These works also reported a strong linear relationship between age and *T*_1,pw_, likely reflecting an increasing pore size due to aging,^32,34^ similar to early characterization of femur specimens with *T*_2_ relaxometry.^35^

It is apparent from all these reports that there is a great deal of variability in *T*_1_ measurements from cortical bone, particularly for measurements specific to PW. To some extent, the intersubject variability in *T*_1,pw_ likely reflects changes in bone porosity (with age or disease), but beyond that the challenges of measuring *T*_1_ with a clinical scanner, even for brain tissue,^36^ are well documented. In cortical bone, this challenge is even greater because of the presence of both BW and PW signals, which need to be distinguished. Gold standard spectroscopic measurements, although they are restricted to ex vivo samples, can potentially clarify the picture.

Independent evaluations of BW and PW *T*_1_ include Horch et al.’s spectroscopic measurements of six human cadaveric femur specimens, reporting *T*_1,bw_ ≈ 350ms and *T*_1,pw_ ≈ 1s at 4.7T.^6^ A follow-up study involving 14 such specimens provided more detail and was the first demonstration of the high variability of PW *T*_1_: *T*_1,bw_ = 340–370ms and *T*_1,pw_ = 380–775ms.^20^ Also using spectroscopic acquisitions, Seifert et al^37^ reported *T*_1,bw_ =320 – 560ms and *T*_1,pw_ = 880 – 1910ms from 15 human cadaveric tibia specimens at 9.4T.

Again, these ex vivo studies make clear the large intersample variability of *T*_1,pw_, but somewhat lost in these works is their demonstration of large intrasample distributions of *T*_1,pw_. From Horch et al,^6^ figure 3 shows that from a single sample *T*_1,pw_ is distributed from ≈ 400 ms to beyond 1s. Similarly, figure 5b from Seifert et al^37^ shows *T*_1,pw_ distributed over a similar, though perhaps slightly longer, range of *T*_1_ (at higher field). Appreciating the distribution of PW *T*_1_ is particularly important for BW imaging methods that aim to suppress PW by inversion-recovery preparation,^20^ and may also affect bicomponent analysis methods^38^ through varying extents of *T*_1_-weighting of the PW signals. Here, we revisit this issue and present both the intersample variation and the intrasample distribution of *T*_1_ of BW and PW from ex vivo samples of human cortical bone.

## 2 | METHODS

### 2.1 | Sample preparation

Cortical bone specimens (*N* = 31, approximate dimensions: 8 mm × 5mm × 2 mm) were extracted from the diaphysis of cadaver radius bones (16 males and 15 females, aged 57–84 years old, mean ± standard deviation (SD): 73 ± 7.4 years) provided by United Tissue Network (Phoenix, AZ, USA). Each specimen was cut such that the osteons were oriented parallel to the long dimension. The donor age range was chosen to correspond to the age range that includes most osteoporotic patients.^39^ The samples were stored in phosphate buffered saline at −20° C before being thawed, and the surface was blotted dry of fluid immediately prior to NMR measurements. Bone volume was measured from each sample using Archimedes’ principle.

### 2.2 | NMR measurements

All NMR measurements were performed on a 4.7T Agilent system with an in-house fabricated low-proton loop-gap style RF coil.^40^ Inside the loop-gap coil, the osteonal axis of the bone specimens was perpendicular with the main magnetic field. A 21.2-μL sample of deionized H_2_O with long *T*_2_ (≈ 2.5 s) was included in all NMR measurements as a volume reference.

To measure *T*_1_ across the full spectrum of bone water *T*_2_ values, an inversion recovery (IR) prepared Carr–Purcell–Meiboom–Gill (CPMG) sequence was used. Each IR period was preceded with a 15-s delay and then involved a ≈ 13.4 μs hard RF inversion followed by one of 15 different inversion delays, log-spaced from 10 to 5000 ms. Each CPMG readout was comprised of 10 000 echoes spaced uniformly from 0.1 to 1000 ms.^6^ Prior to the first and following the last IR-CPMG measurement, the same CPMG acquisitions were run without IR preparation, to provide *T*_2_ measurements of equilibrium magnetization. Signals from four excitations were averaged for each measurement, resulting in a total acquisition time of ≈ 18 min per sample.

### 2.3 | Data processing

All data processing was performed with MATLAB R2022a (The MathWorks, Natick, MA). For each bone, the two equilibrium CPMG acquisitions were averaged and the resulting echo magnitudes were used to estimate a *T*_2_ spectrum, defined here as *S*(*T*_2_). For this calculation, consistent with previous work,^6^ echo magnitudes were fitted to a sum of 128 decaying exponential functions, using non-negative least-squares (NNLS) analysis with minimum curvature regularization.^41^ This spectrum was segmented into three domains of interest: the bound water domain $\left({𝒯}_{bw}:T_{2}=0.15\text{−}1.2\;ms\right)$; the pore water domain $\left({𝒯}_{pw}:T_{2}=1.2\text{−}600\;ms\right)$; and the reference marker domain $\left({𝒯}_{\text{ref}}:T_{2}>0.6\;s\right)$.

The reference marker signal amplitude, *S*_ref_, was computed as the sum of signal over ${𝒯}_{\text{ref}}$. Each *T*_2_ spectrum was then scaled in amplitude as

(1) $$\tilde{S}\left(T_{2}\right)=\frac{C_{\text{ref}}V_{\text{ref}}}{S_{\text{ref}}V_{\text{bone}}}S\left(T_{2}\right),$$

where *V*_bone_ was the measured bone sample volume and *V*_ref_ and *C*_ref_ were the known reference marker volume and proton concentration, respectively. From these scaled spectra, the bound water concentration, *C*_bw_, was computed as the sum of signal over ${𝒯}_{bw}$, and likewise for the pore water concentration, *C*_pw_, over ${𝒯}_{bw}$.

For *T*_1_ − *T*_2_ analysis, the complex echo amplitudes of each of the 15 IR-CPMG readouts were subtracted from the averaged equilibrium CPMG acquisition, resulting in data characterized by decay along both the IR and echo-time domains. These echo magnitudes were then fitted by NNLS to a two-dimensional *T*_1_ − *T*_2_ spectrum (*S*(*T*_1_,*T*_2_)) of 128 decaying exponential functions for *T*_2_ and 65 corresponding functions for *T*_1_.^41,42^ As done with *S*(*T*_2_) in Equation (1), each S(*T*_1_,*T*_2_) was normalized in amplitude and is subsequently referred to as $\tilde{S}\left(T_{1},T_{2}\right)$.

One-dimensional characterizations of *T*_1_ for bound and pore water were generated by summing $\tilde{S}\left(T_{1},T_{2}\right)$ across the bound and pore water *T*_2_ domains, respectively:

(2) $${\tilde{S}}_{x}\left(T_{1}\right)=\sum_{T_{2}\in {𝒯}_{x}}{\tilde{S}}_{x}\left(T_{1},T_{2}\right),$$

where ‘*x*’ indicates either ‘bw’ or ‘pw’. Finally, from the *T*_2_ spectrum and the two *T*_1_ spectra, the geometric mean *T*_1_ and *T*_2_ for both bound and pore water were computed as

(3) $${\bar{T}}_{2,x}=exp\left[\frac{\sum_{T_{2}\in {𝒯}_{x}}log\left(T_{2}\right)\tilde{S}\left(T_{2}\right)}{\sum_{T_{2}\in {𝒯}_{x}}\tilde{S}\left(T_{2}\right)}\right],$$

and

(4) $${\bar{T}}_{1,x}=exp\left[\frac{\sum_{T_{1}}log\left(T_{1}\right){\tilde{S}}_{x}\left(T_{1}\right)}{\sum_{T_{1}}{\tilde{S}}_{x}\left(T_{1}\right)}\right],$$

again with ‘*x*’ indicating either ‘bw’ or ‘pw’.

## 3 | RESULTS

**FIGURE 1:**
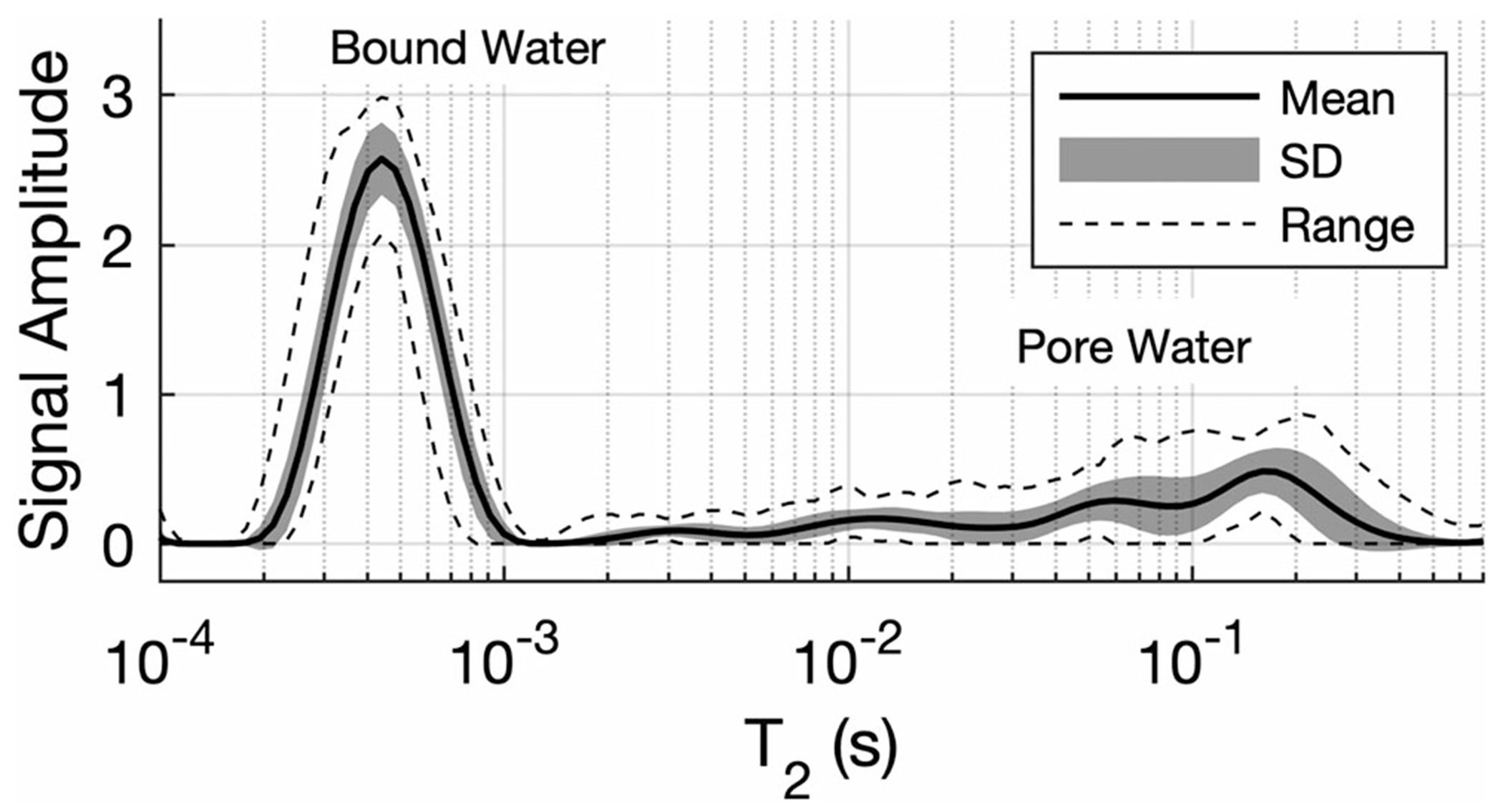
Mean, SD, and range of *T*_2_ spectra measured from 31 bone samples. The *T*_2_ domain has been cropped to show only the bound and pore water domains. The amplitude is in concentration per sample point, meaning that the sum over either PW or BW domains equals *C*_pw_ or *C*_bw_, respectively

**FIGURE 2:**
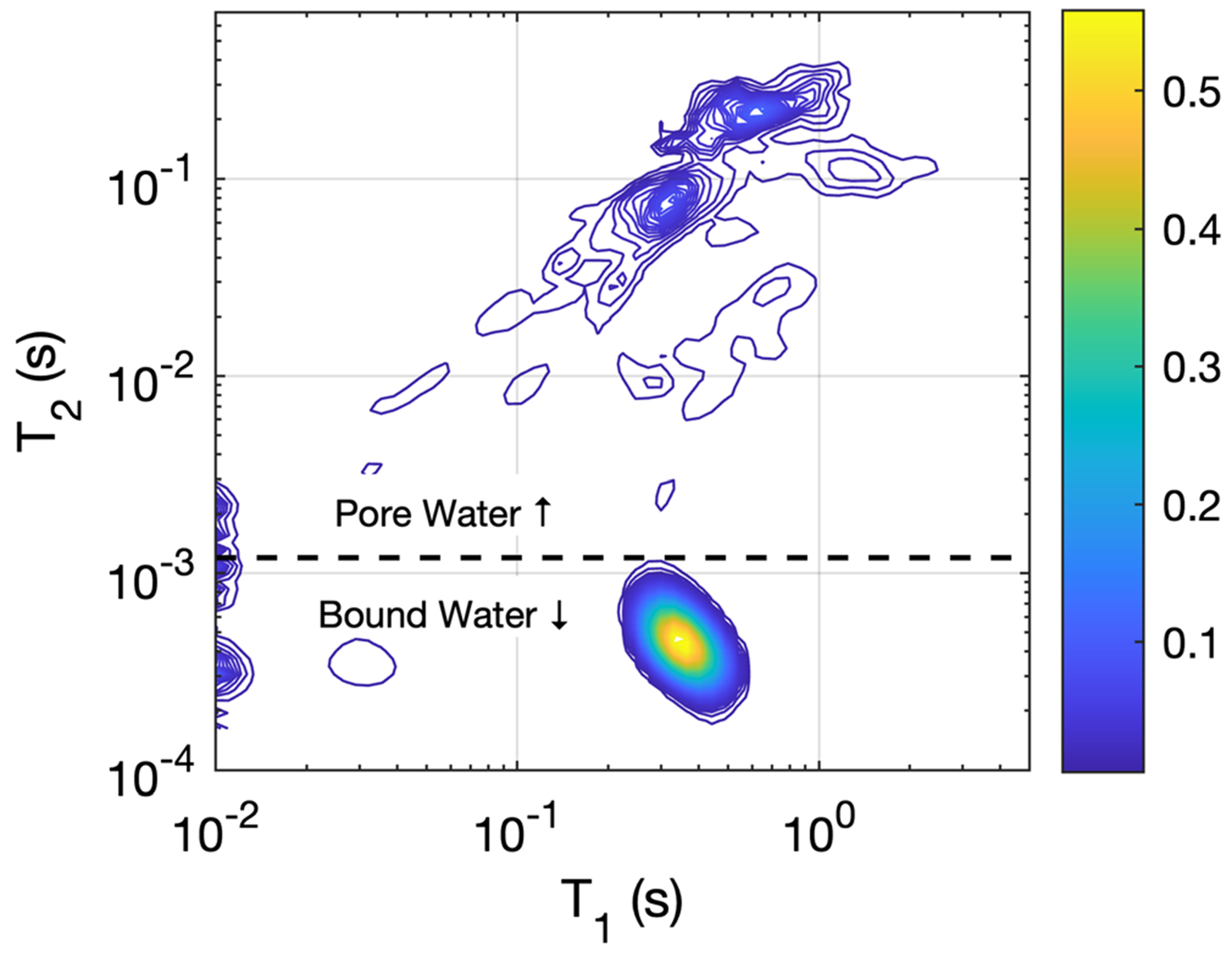
Contour map of the average (across samples) cortical bone water *T*_1_ − *T*_2_ spectrum. The amplitude is in concentration per sample point, meaning that the sum over either PW or BW domains equals *C*_pw_ or *C*_bw_, respectively

Figure 1 shows the mean, SD, and range of the scaled *T*_2_ spectra, $\tilde{S}\left(T_{2}\right)$, derived from all 31 bone samples. The mean ± SD of *C*_bw_ and *C*_pw_ across all samples is reported in Table 1 and values are similar to those reported from previous studies.^6,9^ The average (across all bone samples) of the scaled 2D *T*_1_ − *T*_2_ spectra, $\tilde{S}\left(T_{1},T_{2}\right)$, is presented in Figure 2. Both Figures 1 and 2 have been cropped to show only the bound- and pore-water *T*_2_ domains. In Figure 2, the signals fitted at the lower edge of the *T*_1_ domain were considered artifactual and are not considered in subsequent analysis. This mean spectrum shows that bound water is largely represented by a single, well-defined component, while the pore water signal is distributed widely over both *T*_1_ and *T*_2_.

**FIGURE 3:**
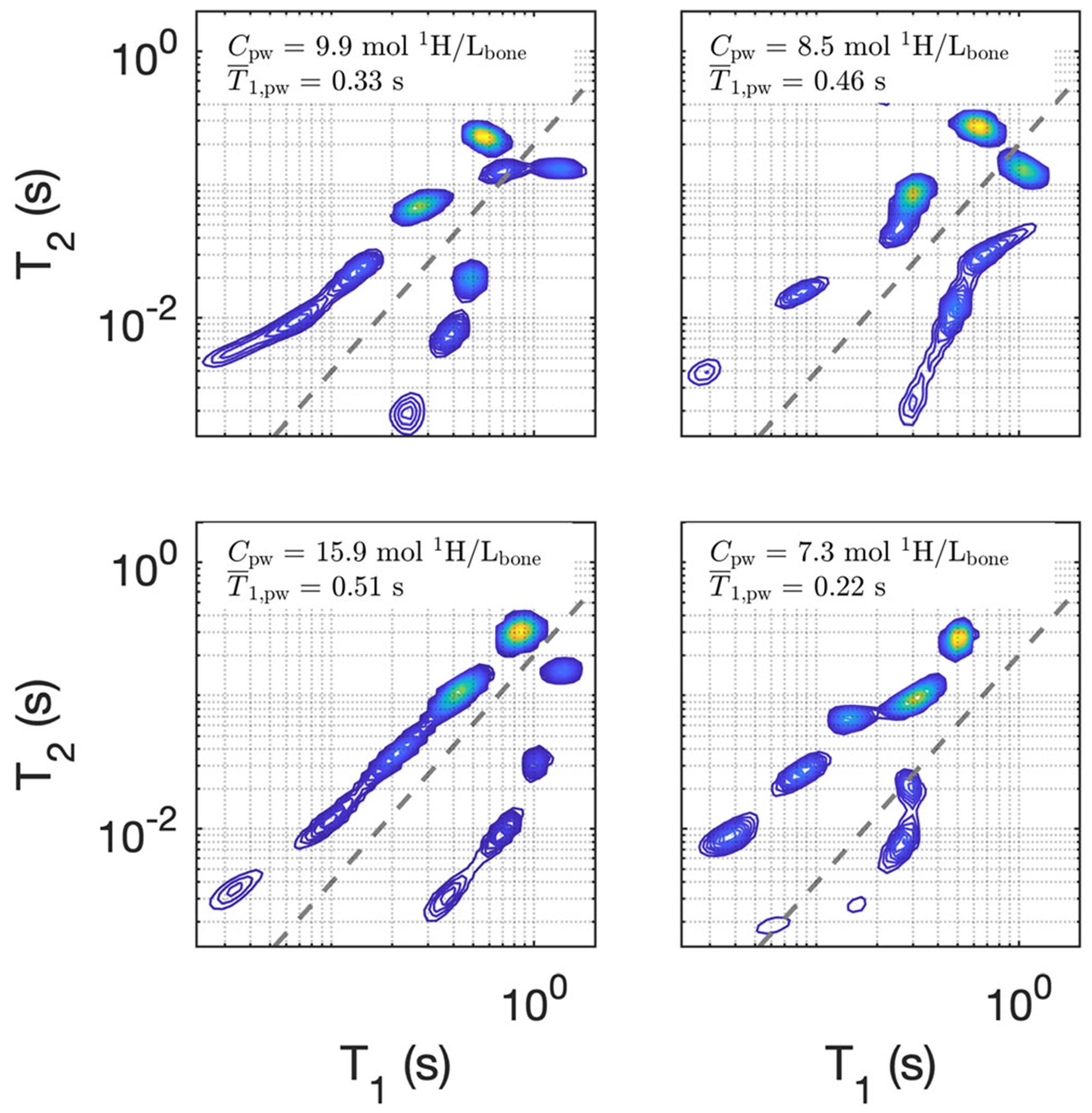
The pore water *T*_1_ − *T*_2_ spectra of four example bone samples, demonstrating both intra- and intersample variations in the pore water relaxation characteristics. Each frame is color-mapped independently, but the sum signal is equal to *C*_pw_, which is shown for each case (along with ${\bar{T}}_{1,pw}$). The gray dashed lines distinguish two signal domains with different *T*_1_ : *T*_2_ characteristics

Figure 3 provides a closer look at the pore water relaxation characteristics from four example bones. These four spectra demonstrate both the intra- and intersample variation in pore water relaxation characteristics. In no case was *T*_1,pw_ close to mono-exponential, and between samples the largest peaks in the spectrum varied in *T*_1_ by hundreds of milliseconds. Interestingly, all samples showed ‘lines’ of signals residing in two different *T*_1_ − *T*_2_ domains. Empirically, these domains can be approximately separated by the power-law equation, $T_{2}=0.2T_{1}^{\left(1.7\right)}$(shown as a gray dashed line on each of the log–log plots in Figure 3).

**FIGURE 4:**
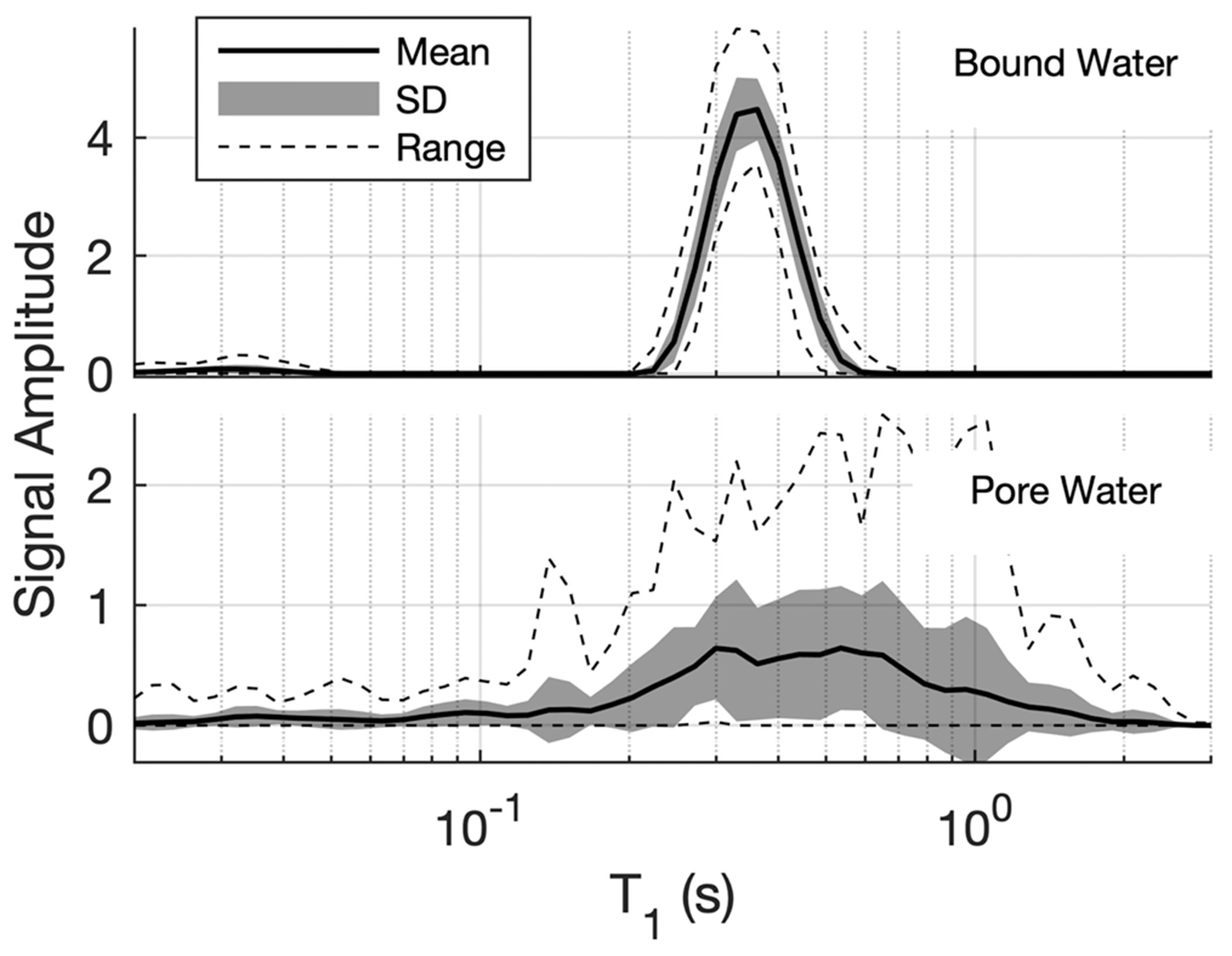
One-dimensional presentations of the mean, SD, and range of bound and pore water *T*_1_ spectra. The amplitude is in concentration per sample point, meaning that the sum over either the PW or BW spectrum equals *C*_pw_ or *C*_bw_, respectively

It is somewhat easier to appreciate the variation of *T*_1_ across all bone samples with 1D spectra, as shown in Figure 4. The top frame shows the mean, SD, and range of ${\tilde{S}}_{bw}\left(T_{1}\right)$, computed across all bone samples, and the corresponding plot for ${\tilde{S}}_{pw}\left(T_{1}\right)$ is shown in the lower frame. It is apparent that the bound water *T*_1_ is primarily mono-exponential and highly reproducible across samples. In contrast, the mean pore water *T*_1_ spectrum spans more than an order of magnitude, and the SD and range data reflect a relatively large variability across samples.

**FIGURE 5:**
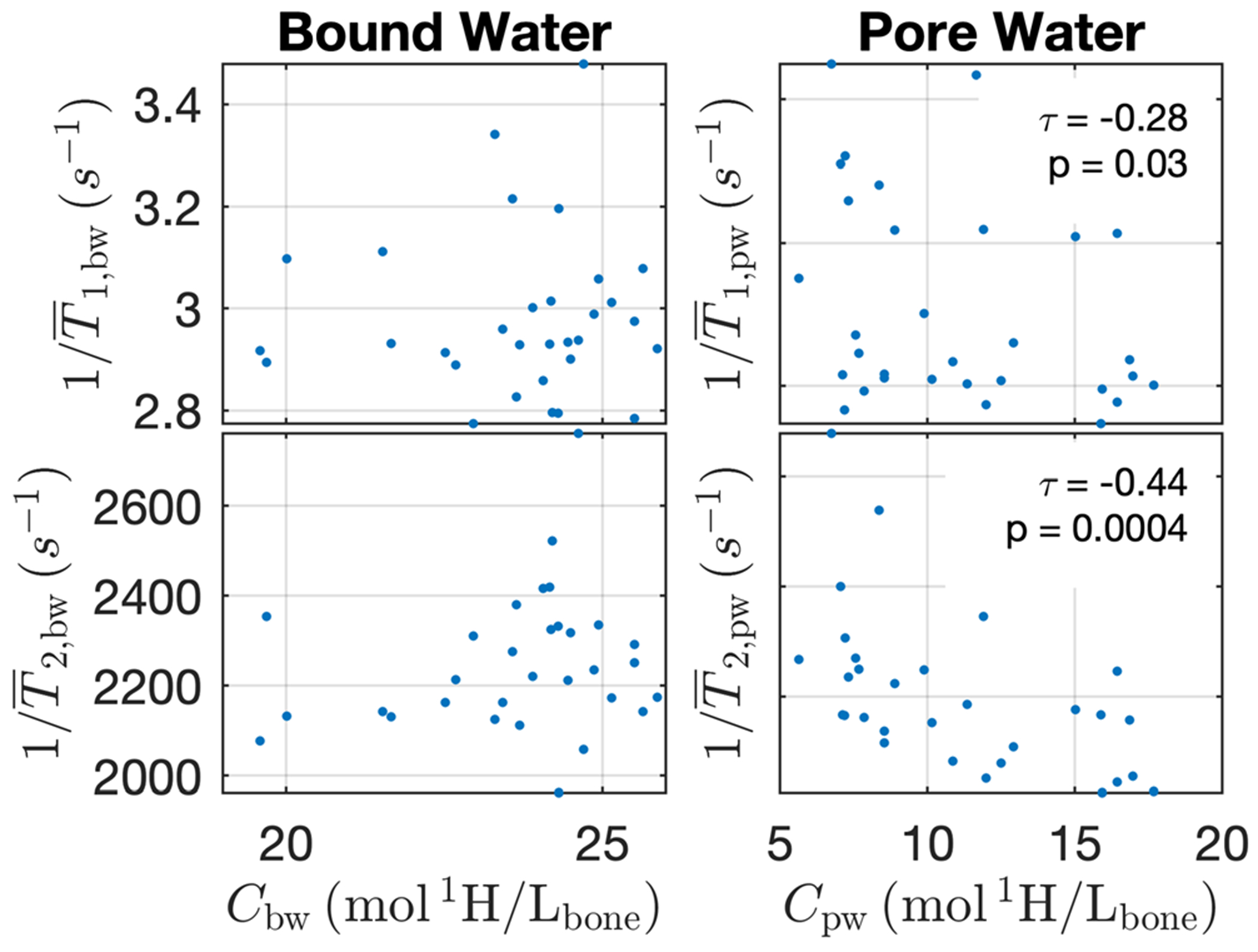
Scatter plots of mean relaxation rates versus bound and pore water concentrations. For pore water, Kendall’s rank correlation coefficients (*τ*) were significant (*p* < 0:05) and are reported along with *p*-values

Finally, for both BW and PW, a summary of concentrations and mean relaxation times is presented in Table 1. Figure 5 presents how these measures relate across bones, showing scatter plots of mean relaxation rate versus concentration, for BW and PW, *T*_1_ and *T*_2_. As expected, there are rank-order relationships between concentration and relaxation rate for PW but not for BW.

## 4 | DISCUSSION

To improve the evaluation of bone fragility, UTE MRI methods have been developed to measure bound, pore, or total water in cortical bone.^10,15,20–22,24,25,29,43,44^ In various ways, each of these methods uses some prior knowledge of the relaxation characteristics of bone water (e.g., to correct for *T*_1_ or *T*_2_ weighting, to define pulse sequence timings, to account for magnetization response to relaxation-selective RF pulses, etc.). While the spectral characteristics of bone water *T*_2_ are relatively well studied and understood,^5,6,20,35,37^ bone water *T*_1_ characteristics remain understudied. This article presents spectral characterizations of water proton *T*_1_ and *T*_2_ from human cadaver cortical bone samples, determined from gold standard spectroscopic measurements. The results provide novel 2D *T*_1_ − *T*_2_ characterizations of bone water, and demonstrate that the *T*_1,pw_ spectrum both is broad within each sample and varies widely between samples.

This study used IR-CPMG data to estimate 2D *T*_1_ − *T*_2_ spectra of radius bone samples (Figure 2). Qualitatively, the mean spectrum in Figure 2 looks similar to previously published 2D spectra from femur^6^ and tibia specimens,^37^ but there are quantitative differences. Similar to the prior work, the results show a BW signal that is essentially mono-exponential in *T*_1_ and *T*_2_, and highly reproducible across individuals. The value of ${\bar{T}}_{1,bw}=336\pm 17\;ms$ here is similar to 357 ± 10 ms reported by Horch et al^6^ from data also collected at 4.7T, while Seifert et al.’s^37^ somewhat greater 480 ± 80 ms came from measurements made at 9.4T.

In contrast to BW, the PW signals reported here covered a wide range of *T*_1_ (*T*_1,pw_ ≈ 100 ms–2 s) and *T*_2_ (*T*_2,pw_ ≈ 1 ms–1 s). Perhaps surprisingly, the lower end of *T*_1,pw_ overlaps with *T*_1,bw_, although qualitatively the same is seen in Horch et al.’s and Seifert el al.’s data. Quantitatively, there is more discrepancy between the *T*_1,pw_ shown here and in those studies. Here, ${\bar{T}}_{1,pw}=387\pm 147\;ms$ is significantly lower than the previous report of 551 ± 120 ms from femur samples at 4.7T,^20^ and much lower than 1210 ± 300 ms from tibia samples at 9.4T.^37^ In a pore system characterized by surface relaxivity, the relaxation rate constant (reciprocal of the time constant) is proportional to the pore surface-to-volume ratio.^45,46^ In cortical bone, faster relaxing signals come from the relatively small lacunae and perhaps canaliculi, while water in the larger Haversian canals relaxes more slowly.^35,47^ The relatively low *T*_1,pw_ here may reflect smaller pore sizes found in the radius compared with those from the tibia and femur.^48^

With increasing age, increasing bone porosity is reflective of a greater volume of large diameter pores.^35^ Given this and the association between pore size and relaxation rates, we expect samples with greater *C*_pw_ to have generally larger sized pores and, therefore, generally longer relaxation time constants. Indeed, Akbari et al. have shown a strong linear correlation between in vivo measures of pore water *T*_1_ in the tibia and age,^32^ and suggested this measure as a surrogate for porosity. The scatter plots shown in Figure 5 show significant rank correlation coefficients (Kendall’s *τ*, *p* < 0:05) for $1/{\bar{T}}_{1,pw}$ and $1/{\bar{T}}_{2,pw}$ with respect to *C*_pw_. However, these relationships, particularly for $1/T_{1}$, are not linear or especially strong and so inference about porosity from geometric mean relaxation rates should be made with caution.

One particularly interesting observation from the PW data here is the presence of signals with substantially different *T*_1_ : *T*_2_ ratios—those above and below the dashed lines in Figure 3. To our knowledge, this has not been previously reported, and may indicate that more than just pore size variation contributes to the varied relaxation characteristics of PW. A detailed investigation of the anatomical origins of these different PW signal components remains for future studies, but it is worth noting that the fraction of signal with relatively large *T*_1_ : *T*_2_ values (to the lower right of the dashed line; mean ± SD = 0.28 ± 0.16) had a strong linear correlation (Pearson’s) with ${\bar{T}}_{1,pw}\left(r=0.64;p=1\times 10^{-4}\right)$, suggesting that it may be related to water in large pores.

In the context of quantitative MRI methods for evaluating BW and/or PW in bone, the highly variable *T*_1,pw_ may be a source of unwanted measurement variability. To investigate, we used the 2D relaxometry data presented here to compute the effect on a few different methods. We previously estimated both *T*_1,pw_ and *T*_1,bw_ to be ≈20% lower at 3T compared with 4.7T,^21^ and so, accordingly, we shifted each of the *T*_1_ spectra measured here down to make these calculations.

For the bicomponent approach,^10,24,25^ which provides measures of the relative amounts of BW and PW, *T*_1_ weighting depends on the excitation flip angle and repetition time (*T*_R_), as in a spoiled gradient echo sequence. Because the method reports only signal fractions, only the relative weighting of BW and PW signals matters. With *T*_R_ = 100 ms and an excitation flip of 10°, both BW and PW are slightly *T*_1_-weighted, resulting in a PW signal fraction error [(measured–ctual)/actual] of only −0.9 ± 1.3% (mean ± SD across samples), and 0.5 ± 0.7% for the BW signal fraction. If the same flip angle is used with *T*_R_ = 10 ms, the errors become −3.3 ±7.4% (range = −15.3 to 11.7%) for the PW fraction and 2.1 ± 3.8% (range = −4.0 to 10.1%) for the BW fraction, which is still small but potentially important.

For DAFP and AIR measurements of PW and BW concentrations, respectively, the effects of *T*_1,pw_ variation are more complicated. Using the signal equations and parameters for 3.0T from Manhard et al,^21^ the PW concentration errors are 1.5 ± 0.6% (range = 0.7–2.8%), while the BW concentration errors are − 8.8 ± 16.6% (range = −37.0 to 23.1%). The effect on PW measurements with DAFP is small, because, similarly to the bicomponent method, the *T*_1_ weighting is small due to the relatively long *T*_R_ (= 400 ms). However the AIR method relies on an IR preparation to null signal from PW, and so variations in *T*_1,pw_ result in the direct addition (or subtraction) of residual PW signal to intended BW signal.

**FIGURE 6:**
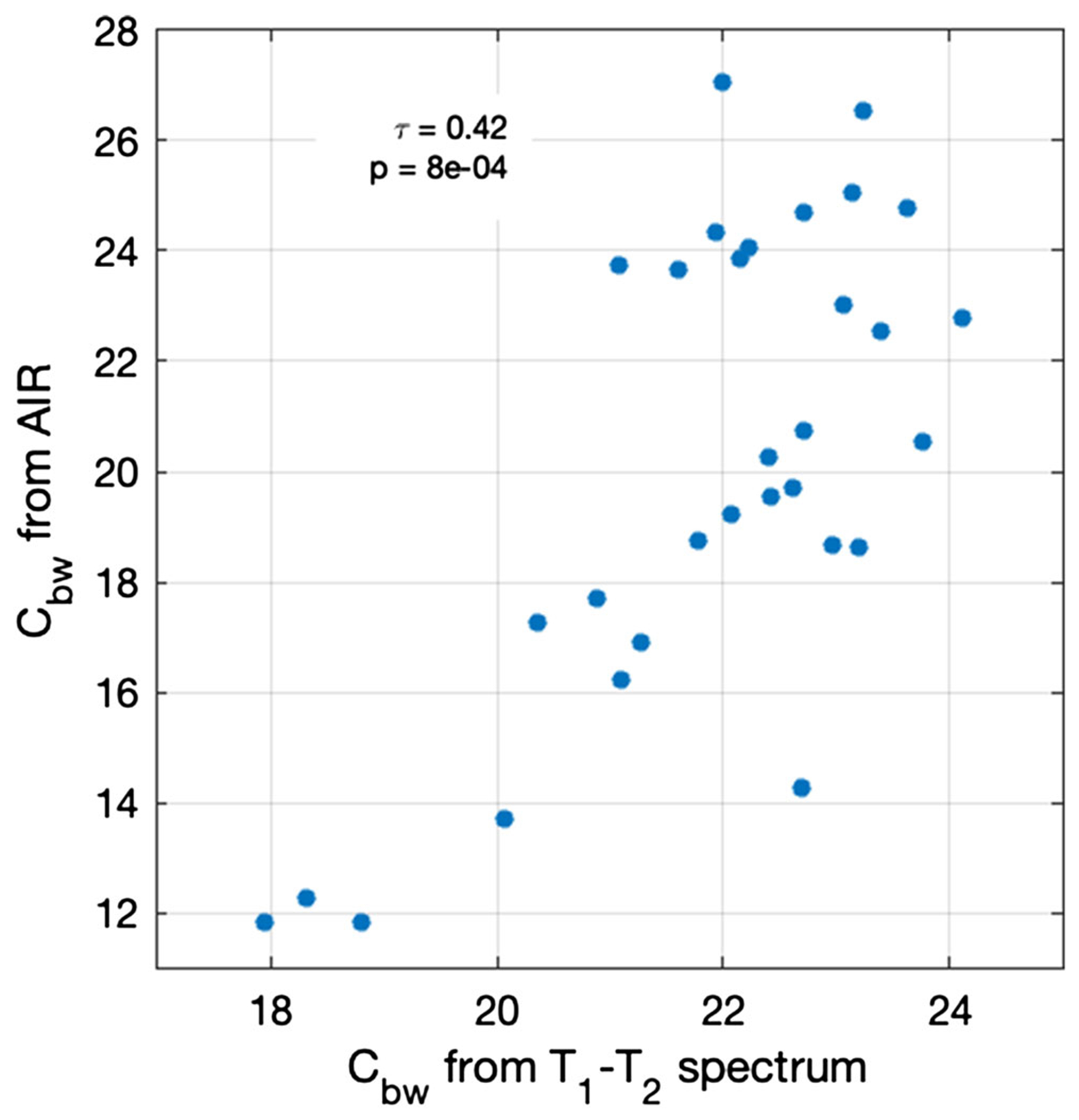
Scatter plot of *C*_bw_ as measured by simulated AIR acquisitions compared with those computed directly from the *T*_1_ − *T*_2_ spectrum

The saving grace for the AIR method is the correlation between *C*_pw_ and ${\bar{T}}_{1,bw}$ (Figure 5). Bones with more PW tend to have relatively long *T*_1,pw_, and in an AIR acquisition this magnetization will be aligned on the negative *Z*-axis after excitation, causing it to subtract from the BW signal. Conversely, in bones with less PW, the residual PW at excitation will tend to add to the BW signal. Because bones with more PW tend to have less BW, and vice versa,^9^ the errors due to imperfect nulling of PW magnetization in AIR will thus tend to cause overestimation of *C*_bw_ in bones with relatively high *C*_bw_ and underestimation of *C*_bw_ in bones with relatively low *C*_bw_. For the bone samples studied here, this effect is shown in Figure 6. Although errors are apparent in the simulated AIR measurements of *C*_bw_, they remain moderately well correlated with the ground truth. Going forward, it may be possible to mitigate the effects of *T*_1,pw_ variation in *C*_bw_ measurements further by using multiple inversion-recovery periods or a subject-specific inversion delay (perhaps based on *C*_pw_ or with an empirically optimized value such as previously done for myelin imaging^49^).

There are several limitations to this study. The sample is small in size (*N* = 31) and from relatively old donors, and the specimens were extracted from closer to the periosteal than the endosteal boundaries of the radius. Also, while most clinical MRI is performed at 1.5 or 3T, the measurements presented here were made at 4.7T and at room temperature. These various limitations will have affected the observed relaxation times, and may have influenced the strength of the relationships between mean relaxation rates and *C*_pw_, but they are unlikely to have caused the broad and highly variable *T*_1,pw_ spectra.

## Figures and Tables

**TABLE 1:** Summary of concentrations and geometric mean relaxation times. Values are mean ± SD computed across samples

	*C* (mol^1^H/*L*_bone_)	${\bar{T}}_{1}(\text{ms})$	${\bar{T}}_{2}(\text{ms})$
Bound water	23.7 ± 1.7	336 ± 17	0.45 ± 0.03
Pore water	11.0 ± 3.9	387 ± 147	54.2 ± 17.4

## Abbreviations:

AIR: adiabatic inversion recovery
BW: bound water
CPMG: Carr–Purcell–Meiboom–Gill
DAFP: double adiabatic full passage
IR: inversion recovery
NNLS: non-negative least-squares
PW: pore wate
SD: standard deviation
UTE: ultrashort echo time
